# Adjustment of the MRSA Search and Destroy policy for outpatients in the Netherlands: a prospective cohort study with repeated prevalence measurements

**DOI:** 10.1186/2047-2994-3-3

**Published:** 2014-01-15

**Authors:** Miranda ML van Rijen, Jan AJW Kluytmans

**Affiliations:** 1 Laboratory for Microbiology and Infection Control, Amphia Hospital, Molengracht 21, PO Box 90158, 4800, RK Breda, The Netherlands; 2 Department of Medical Microbiology and Infection Control, VU Medical Centre, Amsterdam, The Netherlands

**Keywords:** MRSA, Outpatient clinic, Outpatients, *Staphylococcus aureus*, Search & Destroy policy, Transmission

## Abstract

**Background:**

In the Netherlands a successful MRSA Search and Destroy policy is applied in healthcare institutes. We determined the effect of an adjustment in the MRSA Search and Destroy policy for patients in the outpatient clinic on the MRSA transmission to health care workers (HCW).

**Methods:**

In June 2008 an adjustment in the policy for outpatients was introduced in a large teaching hospital. Following this adjustment MRSA positive patients and patients at risk could be seen and treated applying general precautions, without additional protective measures. Also, disinfection of the room after the patient had left was abandoned. To monitor the effect of this policy on the transmission of MRSA all physicians and health care workers of the outpatient clinic were screened for MRSA carriage repeatedly.

**Results:**

Before the introduction of the adjusted policy all physicians and HCW of the outpatient clinic were screened (=0-measurement, n = 1,073). None of them was found to be MRSA positive. After introduction of the policy in June 2008 the screening was repeated in October 2008 (n = 1,170) and April 2009 (n = 1,128). In April 2009 one health care worker was MRSA positive resulting in a mean prevalence of 0.09%. This is lower than the known prevalence in HCW. The health care worker was colonized with the livestock-related *Spa* type t011. As far as we could verify, no patients with this *Spa*-type had been cared for by the health care worker.

**Conclusions:**

The adjusted MRSA policy did not lead to detectable transmission of MRSA to HCW and was associated with less disturbances in the work flow.

## Background

Methicillin-resistant *Staphylococcus aureus* (MRSA) is one of the leading pathogens causing infections in patients admitted to healthcare institutes all over the world. To prevent transmission of MSRA in healthcare institutes, a stringent 'Search and Destroy (S&D) policy’ is applied in the Netherlands and Scandinavian Countries [1]. This policy focuses on isolation of MRSA carriers and of patients at increased risk for MRSA carriage, wearing of personal protective equipment (PPE) by Health Care Workers (HCW), and disinfection of the room after discharge of these patients. The national guideline gives the advice to take these preventive measures for outpatients if it is to be expected that intensive physical contact between HCW and patient would occur during the visit [1]. Intensive contact is specified as 'contact with a significant opportunity for transmission’, for example during undressing of the patient or physical contact with contaminated body sites, like wound inspection. This recommendation is not very easy to apply as it cannot always be predicted what kind of contact will occur.

Traditionally, MRSA was considered to be a healthcare-acquired pathogen (HA-MRSA) and therefore the major risk group were patients that had been admitted to a foreign hospital. However, approximately ten years ago, two new entities emerged. First, in patients without any relationship with healthcare institutes, the so-called Community-acquired MRSA (CA-MRSA) [2,3]. Second, a reservoir for MRSA emerged in animals, in particular in pigs and veal calves, called the livestock-associated MRSA (LA-MRSA) [4-8]. After its emergence, the risk factor 'direct contact with living pigs and veal calves’ was added to the national MRSA guideline in 2006 [1]. The first LA-MRSA in The Netherlands were found in 2003 and by the end of 2011, 39% of all newly identified MRSA strains in humans in the Netherlands already belonged to this variant [9]. This increase of LA-MRSA, which is mainly a problem for hospitals in areas with high pig and/or veal calf densities, results in a considerable disturbance of the process of care on the outpatient clinic, in particular when the patient reports to be at increased risk for MRSA, while he/she is already in the outpatient clinic.

**Figure 1:**
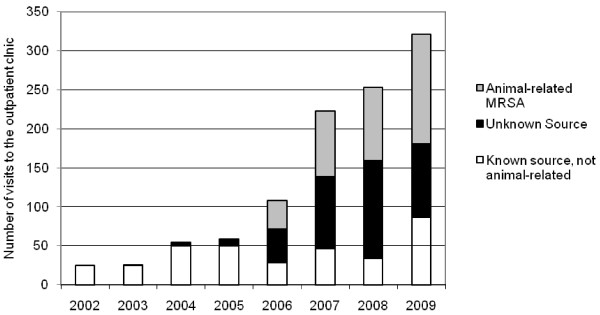
Visits of MRSA positive patients to the outpatient clinic.

The Amphia hospital in the south of the Netherlands has many pig farms in the catchment area (approximately 7000) [10,11]. The flow of patients on the outpatient clinic was mainly hindered by the disinfection of the entire room after the visit of a MRSA positive or patient at risk for MRSA carriage following the national MRSA guideline (a.o. patients that have been admitted to a hospital abroad or have been in contact with pigs/veal calves) [1]. Analysis showed that the major part of the visits from MRSA positive or suspected patients were visits made by patients with contact with pigs/veal calves and by patients without a known risk factor (Figure 1). Due to these logistic problems an adjustment of the policy was considered. It was known that from 2002 until 2006 16 outpatients were unexpectedly found to be MRSA positive by clinical cultures. No infection control measures had been taken while visiting to outpatient clinic. Contract tracing among 211 HCW showed that none of them was found to carry MRSA. Based on this finding we decided to adjust the S&D policy for MRSA positive and outpatients at risk under controlled conditions. Its effect on transmission to HCW was measured by screening all HCW on the outpatient clinic repeatedly.

## Methods

### Content of the adjusted policy and the departments concerned

The adjusted policy for MRSA positive patients and patients at risk for MRSA carriage following the national MRSA guideline was drafted in the infection committee of the hospital and approved by the board of directors. In the adjusted policy, the isolation measures (PPE by HCW) and disinfection of the room after a patient visit were abandoned. Instead of these, the importance of universal precautions was emphasized, i.e. performing hand hygiene, wearing PPE when contact with body fluids and cleaning and disinfection of surfaces contaminated with body fluids or body excreta was anticipated. Besides this, screening of all HCW of the outpatient departments was included in the policy. There were no adjustments in screening policy for patients, i.e. screening of patients at risk (a.o. patients that have been admitted to a hospital abroad or have been in contact with pigs/veal calves) was performed [1]. The Health Inspector was informed about the controlled adjustment in the MRSA policy. In June 2008, the new policy was implemented on all outpatient departments, including departments for functional assessments, radiology and blood sampling.

### Periodic MRSA screening rounds of HCW

To determine transmission to HCW, all HCW on the outpatient departments were sampled periodically. This screening was performed before the implementation of the adjustment (0-measurement) and two times after the implementation, in 2008 and 2009. The target was a compliance of 90 percent of all HCW. Samples were directly plated on chromID *S. aureus* and chromID MRSA agar plates (bioMérieux, La Balme, France), and subsequently placed in a Mueller-Hinton (MH) broth supplemented with 6.5% sodium chloride. The overnight MH broth was subcultured onto both chromID *S. aureus* and chromID MRSA agar Plates [12].

Consequences of eventual MRSA positive HCW were predefined. A history would be taken to identify known risk factors for MRSA carriage [1]. Also, it would be checked whether a MRSA positive patient with the same *Spa* type had been on the department in the period preceding the positive sample. Furthermore, the observed prevalence in the periodic screening will be compared with the prevalence of MRSA in HCW found by Wulf et al., being 0.15 percent [13]. If the percentage of MRSA positive HCW during periodic screening rounds will not exceed this percentage and no contact with MRSA positive patients with the same *Spa* type can be demonstrated, the adjustment in the S&D policy for outpatients would be considered successful.

## Results

### Effect on transmission

In the screening round before implementation of the adjusted policy, there was a response rate of 83% (1073/1296). None of the tested HCW were MRSA-positive. After the introduction of the adjusted policy, the response rates in the two subsequent screening rounds were 83% (1170/1411) and 82% (1128/1376). One HCW from the blood-sampling department was found to be MRSA positive with a livestock-associated *Spa* type t011. No known risk factor following the national MRSA guideline was present [1]. However, she mentioned regular contact with horses and lived in a rural area, which are sometimes mentioned as a risk factor for MRSA carriage [14-16]. All hospital contacts of known patients positive for MRSA t011 (n = 39) were checked for contact with the HCW in the period between negative and positive status (October 2008 and April 2009). During her work she had not been into contact with a patient known to be carrier of this MRSA strain. She was successfully decolonized using mupirocin nasal ointment and chlorhexidine gluconate medicated soap.

## Discussion

The adjusted MRSA policy for outpatients did not result in a demonstrable increase of transmission from patients to HCW. The percentage of MRSA positive HCW found during one of the periodical screening rounds (1/1128=0.09%) was less than the known MRSA prevalence of 0.15% in HCW without known risk factors described by Wulf et al. [13]. This can be explained by the fact that the exposures on the outpatient departments are generally short en less intensive than in the inpatients’ clinic. The universal precautions are probably sufficient to prevent transmission of MRSA in these settings [17].

During the periodical screening MRSA was found in one sample of a HCW. This HCW carried the LA-MRSA type t011. However, she had no contact with pigs or veal calves. During her work, she had not been into contact with a patient known to carry this type. Therefore, it is unknown were she acquired this MRSA. However, she mentioned regular contact with horses and lived in a rural area, which are sometimes mentioned as a risk factor for MRSA carriage [14-16]. Recently, we also showed that regular consumption of poultry and living in a cattle-dense area can be associated with LA-MRSA carriage, so maybe this MRSA was community-acquired [18]. As no exposure had occurred between known MRSA positive patients carrying t011, it can be assumed that the adjustment of the policy did not result in this transmission.

### Limitations

The limitations of this study are threefold. First, the response rate during the periodical screening rounds was 82-83%. Ideally, all HCW have to take samples to exclude any transmission. In theory, a HCW who does not want to take samples, for any reason, can evade all screening rounds. This selective evasion could be a threat because of hidden transmissions. However, all HCW were screened at least once after the introduction of the adjusted policy so we consider this a minor weakness. The second limitation concerns the interval of about 6 months between screening rounds. It is possible that HCW acquired MRSA from a patient and were carrier for a short time, which would result in an omission of this carriage in the periodical screening. Theoretically, this could have resulted in transmission from the HCW to patients. However, if this would lead to ongoing circulation of some MRSA strains this would have been detected in the repeated screenings. Another possible limitation, is the lack of screening of patients that visited the room immediately after a MRSA positive patient. Because we think the nurses and physicians have the most intensive contacts with the patients, they are more at risk to acquire the MRSA from the MRSA positive patients than other patients. Therefore, we screened the HCW and not the patients. However, it can be argued that a patient can also be contaminated by the surroundings instead of by the hands of a HCW. The entire room used to be disinfected after a visit of a MRSA positive patient. In the adjusted policy this was abandoned. However, disinfection of the environment is also a part of the universal precautions. Surfaces that are contaminated with body fluids or body excreta have to be cleaned and disinfected immediately. From our experience, we know that MRSA will mainly be found in dust and not on cleaned, smooth surfaces. In the adjusted policy there is an emphasis on the importance of a clean environment, with a task for both the nurses and the housekeeping. To confirm our findings, we intend to screen some patients that visited the same room after a MRSA positive patient.

Traditionally, the MRSA S&D policy was aimed at a limited risk group, i.e. patients that had been admitted to a hospital abroad. However, the proportion of this risk group has diminished last years because of the rise of other risk groups, mainly patients having direct contact with pigs and/or veal calves and patients without known risk factors (Figure 1) [11,19]. The policy must move along these changing epidemiology. We showed that the described pragmatic approach is a safe way to control MRSA on the outpatient clinic. The results were taken into account by the policy makers to include this adjustment in the national guideline in 2012 [1].

## Conclusions

Our study shows that universal precautions in the outpatient department prevent widespread transmission of MRSA to HCW. The absence of MRSA positive HCW contributes to the final goal of the S&D policy: prevention of MRSA in patients. The adjusted policy is safe for HCW, leads to better logistics on the outpatient clinic and it is a more patient friendly approach.

## Abbreviations

HCW: Health care workers; LA-MRSA: Livestock-associated methicillin-resistant *Staphylococcus aureus*; MRSA: Methicillin-resistant *Staphylococcus aureus*; PPE: Personal protective equipment; S&D: Search and destroy.

## Competing interests

The authors declare that they have no competing interests.

## Authors’ contributions

MvR designed and coordinated the study, gathered en interpreted data, and drafted the manuscript. JK coordinated the study and participated in interpretation of the data. He also helped to draft the manuscript. Both authors read and approved the final manuscript.

## Authors’ information

MvR and JK coordinate and give advice about the implementation of infection control guidelines in the hospital. Furthermore, they conduct multiple studies for methicillin-resistant *S. aureus*.
